# Serum progesterone and prognosis in operable breast cancer.

**DOI:** 10.1038/bjc.1996.292

**Published:** 1996-06

**Authors:** P. E. Mohr, D. Y. Wang, W. M. Gregory, M. A. Richards, I. S. Fentiman

**Affiliations:** Imperial Cancer Research Fund, Clinical Oncology Unit, Guy's Hospital, London, UK.

## Abstract

Several studies have now shown that women with operable breast cancer undergoing tumour excision during the luteal phase of the menstrual cycle have a better prognosis than those having surgery during the follicular phase. As part of a prospective study of prognostic factors in breast cancer, blood was taken at the time of surgery. Between 1975 and 1992 this was available from 289 premenopausal women within 3 days of tumour excision. All were treated by either modified radical mastectomy or breast conservation including axillary clearance and the date of last menstrual period (LMP) was known in 239 (80%) cases. Blood samples were assayed for both oestradiol (E2) and progesterone (P). Because of the wide inter-individual variation in E2 levels there was no clear relationship between E2 and LMP. However, using a running mean smoothing technique the expected cyclical variation could be discerned. There was no significant association between E2 and survival. Smoothing of the P data yielded a pattern similar to the normal hormone profile. Those cases with a progesterone level of 4 ng ml-1 or more had a significantly better survival than those with a level < 4 ng ml-1. This was especially clear in node-positive patients (P < 0.01). The possibility of misclassification of menstrual cycle status, because of misreported LMP, has been minimised by applying an independent hormonal measurement (P) of cycle activity. This parameter will also identify women who may be undergoing anovular cycles. Thus this study has confirmed that a raised level of progesterone at the time of tumour excision is associated with an improvement in prognosis for women with operable breast cancer.


© 1996 Stockton Press All rights reserved 0007-0920/96 $12.00

### Serum progesterone and prognosis in operable breast cancer

PE Mohr[1], DY Wang[2], WM Gregory[1], MA Richards[3] and IS Fentiman[1]

[1]*Imperial Cancer Research Fund, Clinical Oncology Unit, Guy's Hospital, London SE1 9RT;* [2]*Unit of Metabolic Medicine, St Mary's Hospital Medical School, London W2 1PG;* [3]*Sainsbury Department of Palliative Medicine, St Thomas' Hospital, London SE1, UK.*

**Summary** Several studies have now shown that women with operable breast cancer undergoing tumour excision during the luteal phase of the menstrual cycle have a better prognosis than those having surgery during the follicular phase. As part of a prospective study of prognostic factors in breast cancer, blood was taken at the time of surgery. Between 1975 and 1992 this was available from 289 premenopausal women within 3 days of tumour excision. All were treated by either modified radical mastectomy or breast conservation including axillary clearance and the date of last menstrual period (LMP) was known in 239 (80%) cases. Blood samples were assayed for both oestradiol (E2) and progesterone (P). Because of the wide inter-individual variation in E2 levels there was no clear relationship between E2 and LMP. However, using a running mean smoothing technique the expected cyclical variation could be discerned. There was no significant association between E2 and survival. Smoothing of the P data yielded a pattern similar to the normal hormone profile. Those cases with a progesterone level of 4 ng $ml^{-1}$ or more had a significantly better survival than those with a level <4 ng $ml^{-1}$. This was especially clear in node-positive patients ($P<0.01$). The possibility of misclassification of menstrual cycle status, because of misreported LMP, has been minimised by applying an independent hormonal measurement (P) of cycle activity. This parameter will also identify women who may be undergoing anovular cycles. Thus this study has confirmed that a raised level of progesterone at the time of tumour excision is associated with an improvement in prognosis for women with operable breast cancer.

**Keywords:** breast cancer; menstrual phase; progesterone

Remission of advanced breast cancer following oophorectomy in premenopausal women was first reported nearly a century ago (Beatson, 1896). It is now generally accepted that ovarian hormones have an important role in the clinical course of many human breast cancers. A previous study from Guy's Hospital (Badwe *et al.*, 1991) had indicated that the timing of surgery within the menstrual cycle was an important factor in determining both disease-free and overall survival. Prognosis was significantly worse for patients operated on between days 3 and 12 of the cycle, compared with other times. This effect was more pronounced in patients with histologically confirmed axillary nodal metastases but was unrelated to the oestrogen receptor status of the primary cancer.

Although some other centres reported similar findings (Senie *et al.*, 1991; Saad *et al.*, 1994; Veronesi *et al.*, 1994) there have been other negative studies (Powles *et al.*, 1991; Sainsbury *et al.*, 1991; Low *et al.*, 1991). There are major difficulties in comparing these published results partly because of differences in timings used, errors in self-reporting, anovular cycles and possible variations in treatment. Despite this, a recent meta-analysis demonstrated that overall there is a significant effect of timing of surgery on prognosis (Fentiman *et al.*, 1994).

Because all the studies were based upon retrospective data, Badwe *et al.* (1994) attempted to overcome these problems by measuring oestradiol (E2) and progesterone (P) on stored serum from 271 premenopausal patients operated upon between 1975 and 1985. Taking a cut-off of $\geqslant 1.5$ ng $ml^{-1}$ of P there was a significantly better prognosis in node-positive cases with higher P levels. Further blood samples were available from 200 other patients operated on between 1979 and 1992, and concentrations of both E2 and P were assayed in these and combined with the previous results. This report describes the influence of menstrual cycle on the prognosis of an enlarged cohort of 471 premenopausal cases of operable breast cancer.

Correspondence: PE Mohr
Received 12 June 1995; revised 3 January 1996; accepted 16 January 1996

### Material and methods

### *Patients*

Between 1975 and 1992 a total of 1271 premenopausal patients presented to Guy's Hospital Breast Unit with unilateral operable invasive breast cancer. As part of a study of prognostic factors blood had been taken from some of these women. Serum was prepared from the blood and stored at $-20°C$. In a previous report 271 samples were analysed for E2 and P (Badwe *et al.*, 1994). A further search yielded 200 samples from different women, to give an enlarged cohort of 471 cases. Of these 471 women, 289 (61%) had blood taken within 3 days of tumour excision. Thus, an additional 79 evaluable cases were added to this study. All were treated by either modified radical mastectomy or breast conservation therapy comprising tumorectomy, axillary clearance and radiotherapy.

### *Last menstrual period (LMP)*

The date of the LMP, regularity and length of cycle were available from the hospital notes of 239 of the 471 who had blood taken.

### *Blood oestradiol and progesterone assays*

E2 and P were measured using commerically available radioimmunoassay kits (Diagnostic Products) and were similar to those used and described previously (Badwe *et al.*, 1994). The determination of E2 was based on a double antibody radioimmunoassay method using an $^{125}I$-labelled E2 ligand and polyethylene glycol-assisted second antibody to separate bound from free ligand. The assay of P was based on a solid-phase method in which the primary antibody was bound to the walls of plastic tubes. The radioactive P was labelled with $^{125}I$.

To estimate inter- and intra-assay variation, quality control samples from prepared blood sample pools were included in every batch of assays. In addition, 20% of the serum samples were assayed in duplicate. In general the inter- and intra-coefficient of variation was less than 10% for both assays.

*Statistics*

Relapse-free and overall survival were calculated using the method of Kaplan and Meier (1958) and significance of comparisons determined using the log-rank test (Peto *et al.*, 1977). Multivariate analysis was performed using Cox's proportional hazards model (Cox, 1972). A forward stepwise inclusion procedure was used, with a *P*-value for entry of variables of <0.05. Variables considered for the stepwise procedure were age, tumour size, tumour histology (type and grade), axillary nodal status, (number of involved nodes) and serum progesterone.

The smoothing of the E2 and P data was achieved with a running mean method, using the STATA statistical computing package (Computing Resources Center, 1992), where centred subsets of 70 observations were used for calculating each smoothed (mean) value.

### Results

*Progesterone and oestradiol levels and menstrual status*

Of the 461 patients, 289 had blood taken within 3 days of diagnostic excisional biopsy and 234 had known dates of LMP. From the total of 289 patients, 146 (51%) had histologically negative lymph nodes following axillary clearance. The mean perioperative P value of the 289 cases was 3.7 ng $ml^{-1}$ (s.d. = 4.3) with a range of 0.1–20 ng $ml^{-1}$. There were 140 patients with axillary lymph node metastases and the mean P level for this group was 3.6 ng $ml^{-1}$ (s.d. = 3.9). Of the node-positive cases, 99 (71%) received no systemic adjuvant therapy, 22 were given cyclophosphamide, methotrexate and 5-fluorouracil (CMF) and 13 (9%) were given melphalan (L-PAM). The remaining six (4%) were treated by ovarian irradiation with or without prednisolone. The P levels were plotted against the calculated day of the cycle in the 234 women for whom LMP data were available (Figure 1). In spite of the between-person variation in P levels, it is clear that serum P concentration was higher in the luteal phase of the cycle.

Smoothing of the data showed a cyclical change in serum P similar to the expected pattern (Figure 1). Of the 76 patients who were putatively in the first 12 days of the cycle, 92% had P levels of 4 ng $ml^{-1}$ or less. In the 158 cases calculated to be in the luteal phase (day 13 to day 30) 56% had P levels in excess of 4 ng $ml^{-1}$. The value of 4 ng $ml^{-1}$ was chosen by inspection of Figure 1, to put as high a proportion of values from the 0–12 day-in-cycle group into the low/normal progesterone group without going beyond the supposed normal range.

Whereas P levels conveniently help to divide the menstrual cycle into a luteal and a non-luteal phase, E2 on the other hand, has a much more complex pattern (Figure 2). The E2 data, whether smoothed (Figure 2) or not, were not useful in helping to assign the menstrual cycle status of patients.

**Figure 1** Progesterone (P) levels for 239 patients with known date of last menstrual period (LMP), with smoothed progesterone data.

*Progesterone levels and prognosis*

Comparison of patients having P levels >1.5 ng $ml^{-1}$ with those cases with blood levels lower than this showed that there was no significant difference in survival (Figure 3). However, when cases were dichotomised on the basis of P levels of ⩽4 ng $ml^{-1}$ and >4 ng $ml^{-1}$ it was found that those with the higher P concentration had a significantly better survival, as shown in Figure 4 ($P$=0.04). The improved survival was particularly pronounced in the group with nodal involvement and P levels >4 ng $ml^{-1}$ ($P$=0.01; Figure 5). Survival in relation to absolute level of progesterone was also compared for patients who had surgery at both high- and low-risk times as determined by LMP data. There was no direct relationship between survival of patients in the low-risk group based on progesterone level, nor in the high-risk group.

Table I shows the significant prognostic variables that emerged from the multivariate analysis. The major prognostic factors for survival in the entire group were, firstly, number of involved nodes (RR = 2.38, $P$<0.0001); secondly, tumour type (RR = 2.89, $P$<0.0001) and thirdly, serum progesterone (RR = 1.76, $P$=0.027). All were significant for node-positive cases but only tumour type (RR = 3.45, $P$=0.008) was significant in node-negative patients.

**Figure 2** Oestradiol (E2) values for 239 patients with known date of last menstrual period (LMP), with overlying smoothed data.

**Figure 3** Overall survival of patients based on serum progesterone (at time of surgery) ⩽1.5 ng $ml^{-1}$ and >1.5 ng $ml^{-1}$.

**Figure 4** Overall survival of patients based on progesterone $\leqslant$4 ng ml$^{-1}$ and >4 ng ml$^{-1}$.

**Figure 5** Overall survival of node-positive patients based on progesterone $\leqslant$4 ng ml$^{-1}$ and >4 ng ml$^{-1}$.

**Table I** Multivariate analysis showing significant prognostic variables

| | *All patients* (n = 289) *RR (95% CI)* | *Node positive* (n = 140) *RR (95% CI)* | *Node negative* (n = 146) *RR (95% CI)* |
|---|---|---|---|
| Node no.[a] | 2.38 (1.83–3.09) | 2.25 (1.33–3.8) | – |
| *P* | <0.0001 | <0.01 | – |
| Histology[b] | 2.89 (1.97–4.26) | 2.82 (1.78–4.48) | 3.45 (1.63–7.32) |
| *P* | <0.001 | <0.0001 | 0.008 |
| Progesterone[c] | 1.76 (1.04–2.96) | 2.22 (1.15–4.29) | 1.07 (0.44–2.58) |
| *P* | 0.027 | 0.0002 | 0.88 |

[a]Categorised 0 *vs* 1–3 *vs* $\geqslant$4 (three patients were omitted because the number of involved nodes was unknown). [b]Categorised according to Bloom and Richardson grade with non-ductal types coded as grade II. [c]Categorised as $\leqslant$4 ng ml$^{-1}$ *vs* >4 ng ml$^{-1}$. Age and tumour size were also considered as possible explanatory variables, but were not significant in any of the analyses.

### Discussion

We have reported previously that women with operable breast cancer undergoing surgery in the luteal phase of the menstrual cycle have a better prognosis than those operated upon at other times (Badwe *et al.*, 1991). This finding has been confirmed by others but not by all (Fentiman and Gregory, 1993). More recently, the Milan group reported a similar finding on a large series of patients (Veronesi *et al.*, 1994). The aim of this study was to explore more deeply this phenomenon by assessing corpus luteum activity and outcome. Firstly, the accuracy of self-reported LMP has been assessed, together with the possible importance of progesterone as a key agent in influencing prognosis.

The main finding of this study was that premenopausal patients who underwent tumour excision when their blood level of progesterone was >4 ng ml$^{-1}$ had a significantly better overall survival than those treated at a time when P levels were $\leqslant$4 ng ml$^{-1}$. This effect was seen in all cases but was most evident in those with axillary nodal involvement. Within the high- and low-risk times there did not appear to be a dose–response effect of level of progesterone on prognosis. Whether progesterone *per se* is the reason for this finding cannot be ascertained from the present data. Possible mechanisms remain speculative.

The increase in proliferation of human breast cells in the luteal phase of the cycle is intriguing (Anderson *et al.*, 1982; Masters *et al.*, 1977), and the normal peritumoral cells might be exerting an influence on the malignant cells. This would be supported by the observation in our original report that the effect of timing of surgery was equally large in patients with oestrogen receptor-positive and oestrogen receptor-negative tumours.

There is no evidence that there is a causal link between mitotic activity and rise in level of blood P, and indeed progesterone might be acting as a down-regulator of cell proliferation since in endometrial tissue it has been shown that 17$\beta$-hydroxy-dehydrogenase is down-regulated by P. This increases the conversion of E2 to the less biologically active oestrone (E1), while at the same time decreasing the amount of oestradiol receptor and RNA message (Alexander *et al.*, 1990; Mandelond *et al.*, 1991).

Finally, progestogens have been reported to down-regulate IGF-1 RNA message and content in breast cancer cell lines (Papa *et al.*, 1991). If progesterone is the active agent then it might be that the concentration of this steroid is important and it would be predicted that patients treated in the mid-luteal phase, in which values of 20 ng ml$^{-1}$ occur, would have a better prognosis. This was the case in our first study in that when mortality rates were plotted against day of surgery, the lowest death rate was seen between days 18 and 20 (Badwe *et al.*, 1991).

In a previous study we used a progesterone level of 1.5 ng ml$^{-1}$ as cut-off between luteal and follicular phases. However, in this larger study it was found that an unacceptable proportion of women had P values greater than 1.5 ng ml$^{-1}$ even though they were, based on LMP, in the follicular stage of their cycle. Conversely, 42% of patients who were putatively in the luteal phase, based on LMP, had P levels <1.5 ng ml$^{-1}$. One reason for this discrepancy in the latter half of the cycle may be because in 65 cases the exact cycle length was unknown and assumed to be 28 days. Thus, some women with low progesterone levels may have been in the early follicular stage of their next cycle. The chance of laboratory errors is always possible but the inclusion of routine quality controls and replicate samples in every assay batch makes this unlikely. Another possible explanation is the occurrence of anovular cycles. Based on either blood progesterone or urinary pregnanediol levels the incidence of anovulatory cycles has been estimated to be in the region of 25%. Thus Wathen *et al.* (1984) reported that 9/39 (23%) of 'normal' women of reproductive age were experiencing anovular cycles. Despite the apparent discrepancy between LMP and P data, the classical menstrual profile of progesterone was seen after the data were 'smoothed'. This analytical technique also minimises the large between-person variation in levels and the use of single blood specimens. This result indicates that, in general, the LMP and progesterone data are in accord but supports the view that knowledge of both is necessary for establishing that patients are in the luteal phase of the cycle.

Although the oestradiol data showed a classical cyclical pattern after smoothing, there was a very large amount of 'noise' in these data with variations between patients swamping the cycle effect. Thus, not surprisingly, there was no discernible relationship between E2 levels and prognosis. Unlike progesterone, E2 levels cannot therefore be used to categorise patients according to menstrual cycle status, other than the small proportion of mid-cycle cases with very high levels.

The beneficial effect associated with elevated progesterone levels at the time of surgery supports the hypothesis that modification of oestradiol activity by progesterone at the time of surgery can significantly influence the prognosis of some patients with early breast cancer. The mechanism has yet to be elucidated but could have a profound impact on the future treatment of operable breast cancer.

**Acknowledgement**
DYW is grateful to the Breast Cancer Research Fund for financial support.

### References

ALEXANDER IE, SHINE J AND SUTHERLAND RL. (1990). Progestin regulation of estrogen receptor messenger RNA in human breast cancer cell lines. *Mol. Endocrinol.*, **4**, 821-828.

ANDERSON TJ, FERGUSON JP AND RAAB GM. (1982). Turnover in the 'resting' breast: influence of parity, contraceptive pill, age and laterality. *Br. J. Cancer*, **46**, 376-382.

BADWE RA, GREGORY WM, CHAUDARY MA, RICHARDS MA, BENTLEY AE, RUBENS RD AND FENTIMAN IS. (1991). Timing of surgery during menstrual cycle and survival of premenopausal women with operable breast cancer. *Lancet*, **37**, 1261-1264.

BADWE RA, WANG DY, GREGORY WM, FENTIMAN IS, CHAUDARY MA, SMITH P, RICHARDS MA AND RUBENS RD. (1994). Serum progesterone at the time of surgery and survival in women with premenopausal operable breast cancer. *Eur. J. Cancer*, **30A**, 445-448.

BEATSON G. (1896). On the treatment of inoperable cases of carcinoma of the mammae. Suggestions for a new method of treatment. With illustrative cases. *Lancet*, **2**, 104-122.

COMPUTING RESOURCE CENTER. (1992). STATA Reference Manual: Release 3, vol. 2, 5th ed., pp. 361-364. Santa Monica, CA.

COX DR. (1972). Regression models and life tables. *J. R. Stat. Soc.*, **34**, 197-220.

FENTIMAN IS AND GREGORY WM. (1993). The hormonal milieu and prognosis in operable breast cancer. *Cancer Surveys*, **18**, 149-163.

FENTIMAN IS, GREGORY WM AND RICHARDS MA. (1994). Effect of menstrual phase on surgical treatment of breast cancer. *Lancet*, **344**, 402.

KAPLAN EL AND MEIER P (1958). Nonparametric estimation from incomplete observations. *Am. Stat. Assoc. J.*, **53**, 457-481.

LOW SG, GALEA MH AND BLAMEY R. (1991). Timing breast surgery. *Lancet*, **338**, 691.

MANDELOND T, LAVAUD P, SALAZAR G, LAFFARQUE J AND ROCHEFORT H. (1991). Progestins depress estrogen receptor but not cathepsin D level in needle aspirates of benign breast disease. *Breast Cancer Res. Treat.*, **19**, 95-102.

MASTERS JRW, DRIFE JR AND SCARISBRICK JJ. (1977). Cyclic variation of DNA synthesis in human breast epithelium. *J. Natl. Cancer Inst.*, **58**, 1263-1265.

PAPA V, HARTMANN KKP, ROSENTHAL SM, MADDUX BA, SIITERI PK AND GOLDFINE ID. (1991). Progestins induce down-regulation of insulin-like growth factor 1 (IGF-1) receptors in human breast cancer cells: potential autocrine role of IGF-1. *Mol. Endocrinol.*, **5**, 709-717.

PETO R, PIKE MC, ARMITAGE P, BRESLOW NE, COX DR, HOWARD V, MANEL N, McPHERSON K, PETO J AND SMITH PG. (1977). Design and analysis of clinical trials requiring prolonged observation of each patient: II. Analysis and examples. *Br. J. Cancer*, **35**, 1-39.

POWLES TJ, ASHLEY SE, NASH AG, TIDY A, GAZET J-C AND FORD HT. (1991). Timing of surgery for breast cancer. *Lancet*, **337**, 1604.

SAAD Z, BRAMWELL V, DUFF J, GIROTTI M, JORY T, HEATHCOTE I, TURNBULL I, GARCIA B AND STITT L. (1994). Timing of surgery in relation to menstrual cycle in premenopausal women with operable breast cancer. *Br. J. Surg.*, **81**, 217-220.

SAINSBURY R, JONES M, PARKER D, HALL R AND CLOSE H. (1991). Timing of surgery for breast cancer. *Lancet*, **338**, 392.

SENIE RT, ROSEN PP AND RHODES P. (1991). Timing of breast cancer excision during the menstrual cycle influence duration of disease-free survival. *Ann. Intern. Med.*, **115**, 337-342.

VERONESI U, LUINI A, MARIANI L, DEL VECCHIO M, ALVEZ D, ANDREOLI C, GIACOBONE A, MERSON M, PACETTI G, RASELLI R AND SACCOZZI R. (1994). Effect of menstrual phase on surgical treatment of breast cancer. *Lancet*, **343**, 1544-1546.

WATHEN NC, PERRY L, LILFORD RJ AND CHARD T. (1984). Interpretation of single progesterone measurement in diagnosis of ovulation and defective luteal phase: observation on analysis of the normal range. *Br. Med. J.*, **288**, 7-9.


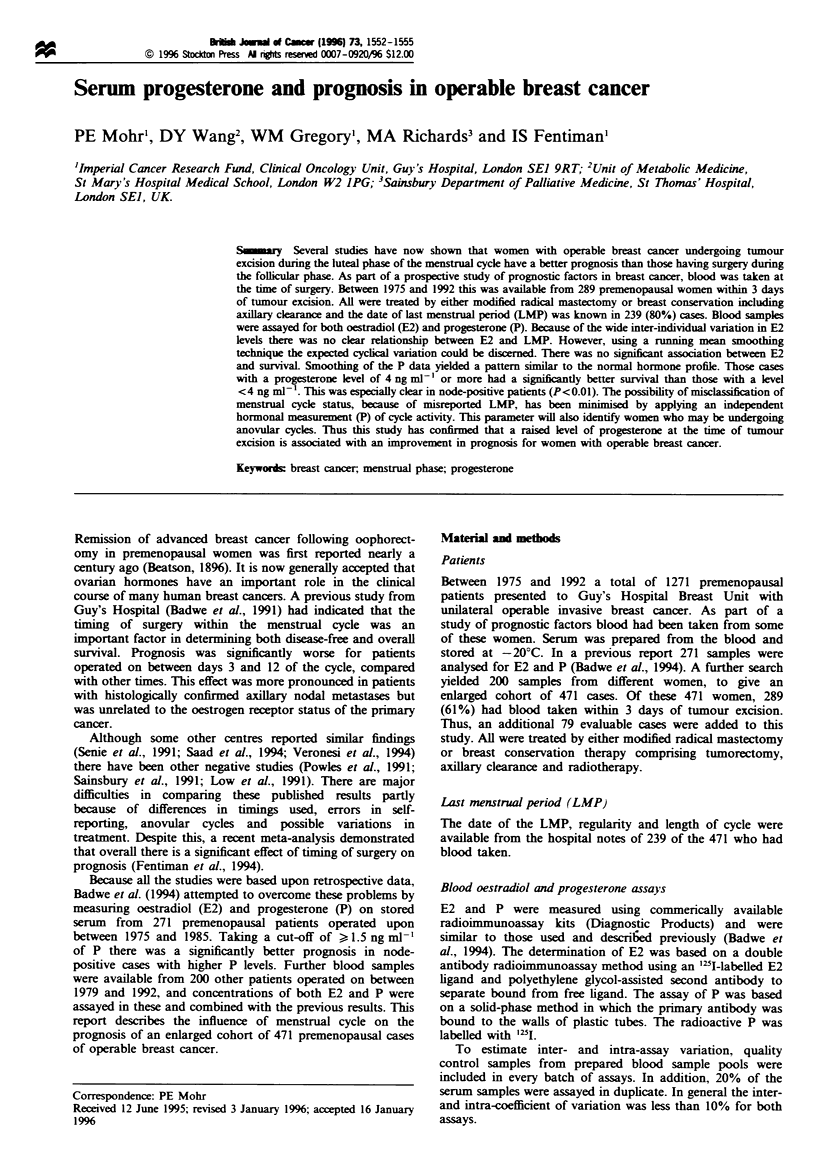

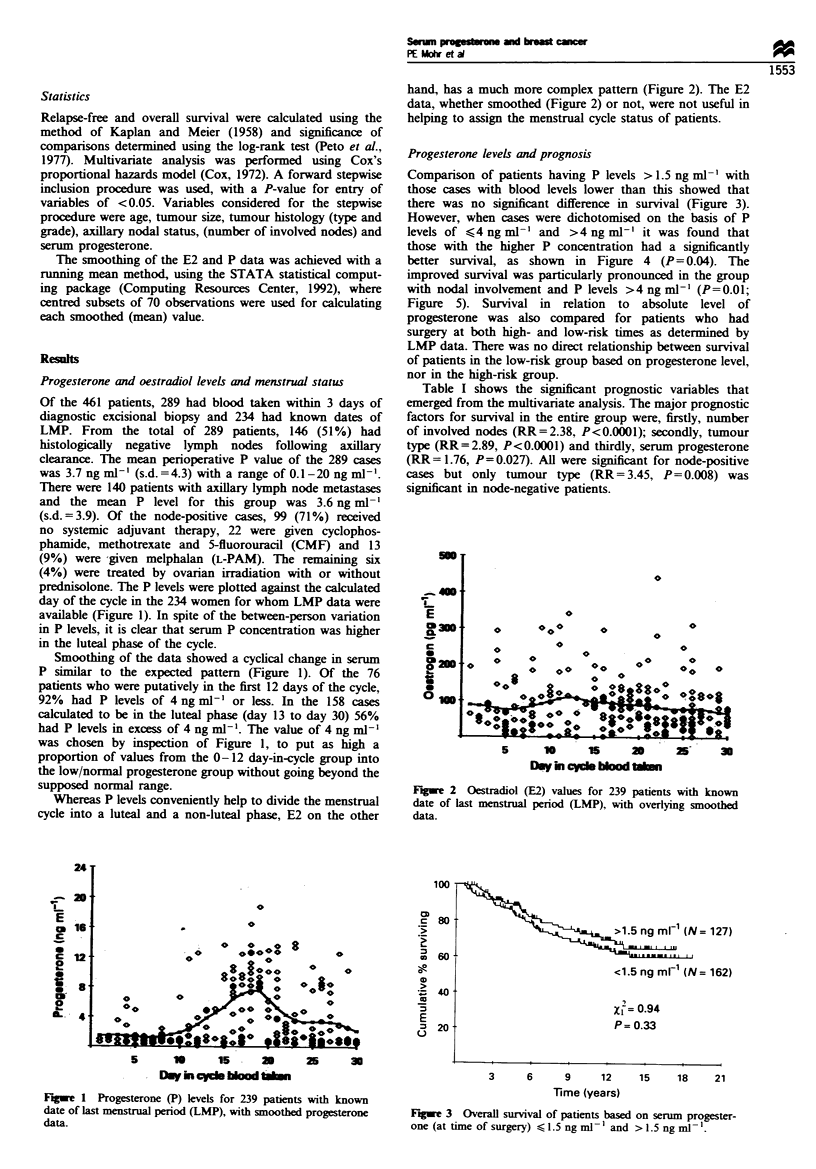

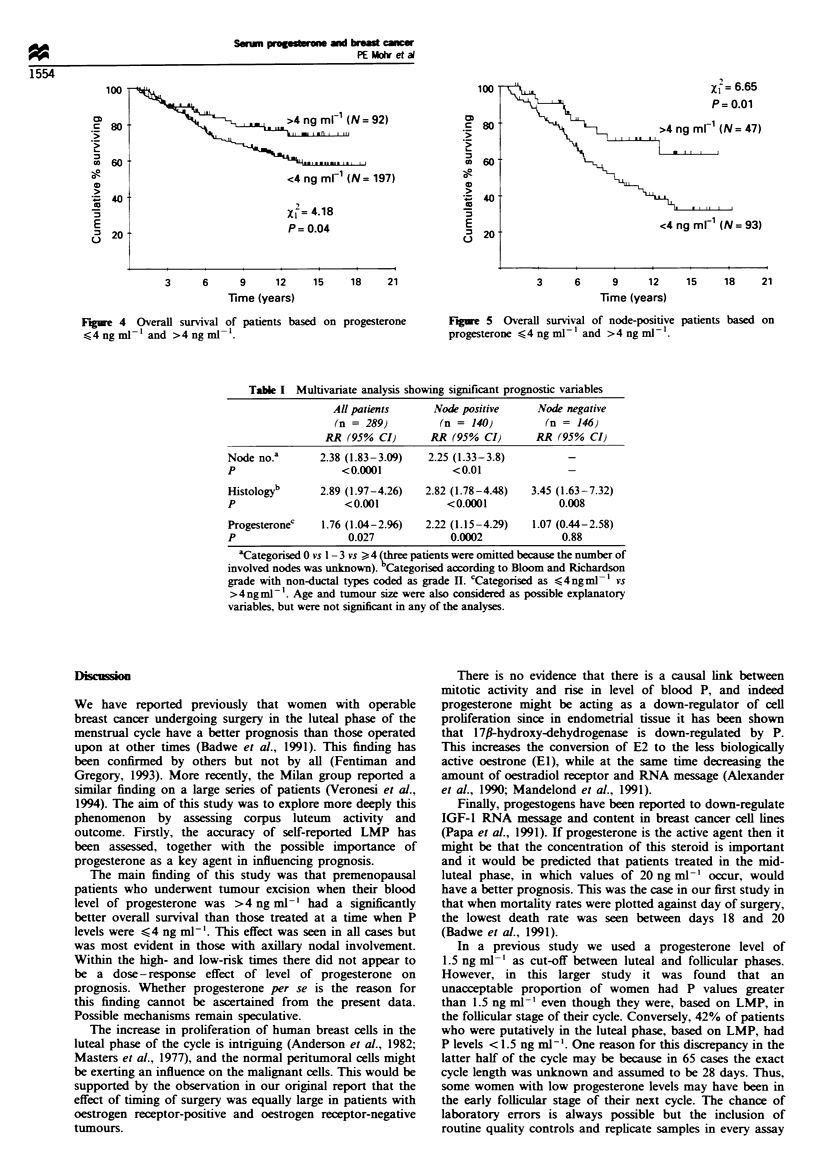

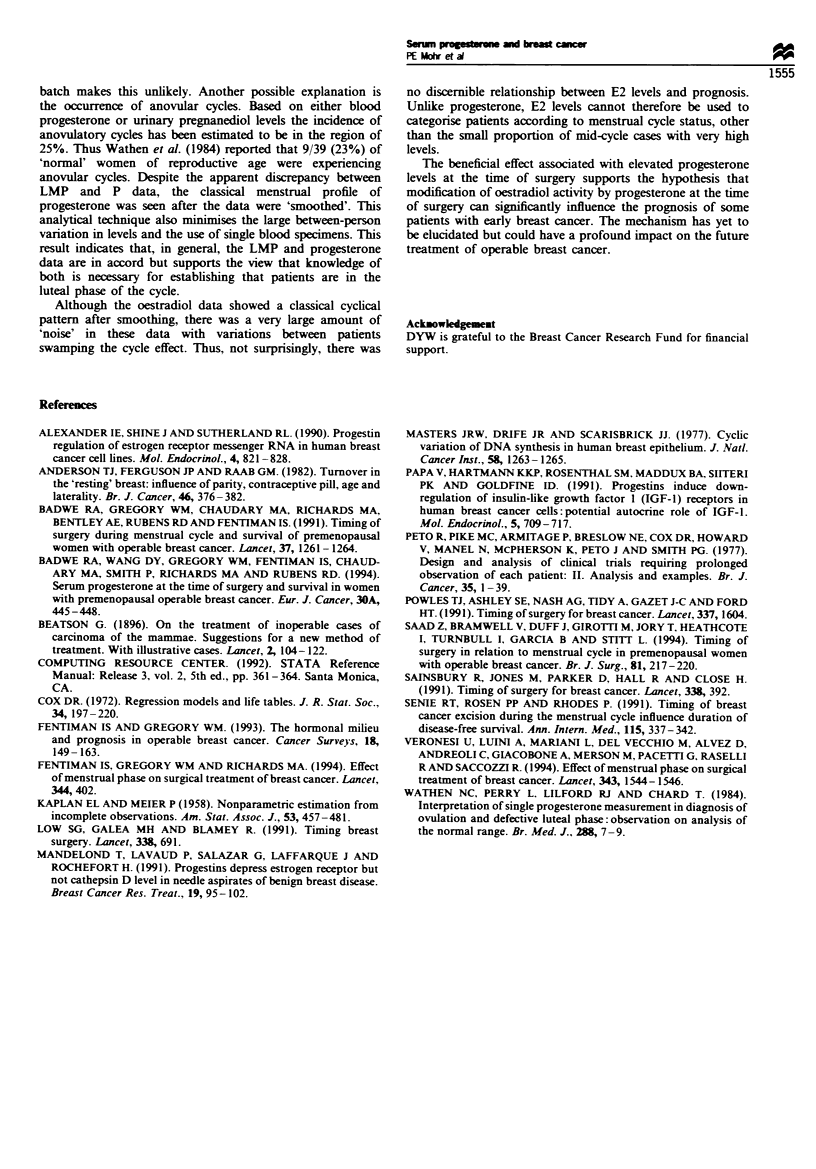


## References

[OCR_00492] Alexander I. E., Shine J., Sutherland R. L. (1990). Progestin regulation of estrogen receptor messenger RNA in human breast cancer cells.. Mol Endocrinol.

[OCR_00499] Anderson T. J., Ferguson D. J., Raab G. M. (1982). Cell turnover in the "resting" human breast: influence of parity, contraceptive pill, age and laterality.. Br J Cancer.

[OCR_00504] Badwe R. A., Gregory W. M., Chaudary M. A., Richards M. A., Bentley A. E., Rubens R. D., Fentiman I. S. (1991). Timing of surgery during menstrual cycle and survival of premenopausal women with operable breast cancer.. Lancet.

[OCR_00511] Badwe R. A., Wang D. Y., Gregory W. M., Fentiman I. S., Chaudary M. A., Smith P., Richards M. A., Rubens R. D. (1994). Serum progesterone at the time of surgery and survival in women with premenopausal operable breast cancer.. Eur J Cancer.

[OCR_00536] Fentiman I. S., Gregory W. M., Richards M. A. (1994). Effect of menstrual phase on surgical treatment of breast cancer.. Lancet.

[OCR_00529] Fentiman I. S., Gregory W. M. (1993). The hormonal milieu and prognosis in operable breast cancer.. Cancer Surv.

[OCR_00555] Masters J. R., Drife J. O., Scarisbrick J. J. (1977). Cyclic Variation of DNA synthesis in human breast epithelium.. J Natl Cancer Inst.

[OCR_00549] Maudelonde T., Lavaud P., Salazar G., Laffargue F., Rochefort H. (1991). Progestin treatment depresses estrogen receptor but not cathepsin D levels in needle aspirates of benign breast disease.. Breast Cancer Res Treat.

[OCR_00558] Papa V., Hartmann K. K., Rosenthal S. M., Maddux B. A., Siiteri P. K., Goldfine I. D. (1991). Progestins induce down-regulation of insulin-like growth factor-I (IGF-I) receptors in human breast cancer cells: potential autocrine role of IGF-II.. Mol Endocrinol.

[OCR_00565] Peto R., Pike M. C., Armitage P., Breslow N. E., Cox D. R., Howard S. V., Mantel N., McPherson K., Peto J., Smith P. G. (1977). Design and analysis of randomized clinical trials requiring prolonged observation of each patient. II. analysis and examples.. Br J Cancer.

[OCR_00578] Saad Z., Bramwell V., Duff J., Girotti M., Jory T., Heathcote G., Turnbull I., Garcia B., Stitt L. (1994). Timing of surgery in relation to the menstrual cycle in premenopausal women with operable breast cancer.. Br J Surg.

[OCR_00587] Senie R. T., Rosen P. P., Rhodes P., Lesser M. L. (1991). Timing of breast cancer excision during the menstrual cycle influences duration of disease-free survival.. Ann Intern Med.

